# Load-Capacity-Constrained Arm-Angle Planning for a Centrally Driven Humanoid Robotic Arm

**DOI:** 10.3390/biomimetics11080592

**Published:** 2026-08-19

**Authors:** Zhongyue Lu, Zhichao Zhu, Shanjun Chen, Tao Jiang, Zirong Luo

**Affiliations:** 1 College of Intelligence Science and Technology, National University of Defense Technology, Changsha 410073, China; luzhongyue@nudt.edu.cn (Z.L.);; 2 National Key Laboratory of Equipment State Sensing and Smart Support, National University of Defense Technology, Changsha 410073, China; 3 School of Automation Science and Electrical Engineering, Beihang University, Beijing 100191, China

**Keywords:** humanoid robotic arm, arm angle, redundancy resolution, load capacity, energy-efficient planning

## Abstract

This paper presents a load-capacity-constrained arm-angle planning method for a centrally driven humanoid robotic arm. Joint-range and singularity constraints are projected into the one-dimensional arm-angle domain to form a geometric feasible set. A static load-capacity constraint is derived from gravity torque, the Jacobian-transpose mapping of a known endpoint load, and individual actuator-torque limits, and is projected into the same domain. Continuous arm-angle values are then selected along a prescribed Cartesian path within the intersection of the geometric and mechanical feasible sets. In a heavy-load simulation, the maximum output-power metric decreased by 13.3%, and the energy value decreased from 30.60 J to 22.95 J (25.0%). In a prototype proof-of-principle test with a 13 N payload (approximately 1.33 kg), each trajectory was executed three times; the controller-recorded shoulder peak was approximately 1.83% lower, and the recorded energy value decreased from 30.378 J to 28.85 J (5.03%). The prototype values are descriptive because run-level statistics and measurement uncertainty are unavailable, and the experiment covers only one payload and one path. The results support the proposed static, load-capacity-aware planning principle for the tested slow-motion conditions but do not establish general performance or real-time suitability.

## 1. Introduction

Humanoid robotic arms must combine human-like reachability with practical requirements on payload, energy consumption, and safety. This requirement is increasingly important as recent humanoid and mobile-manipulation systems move toward loco-manipulation, shared control, flexible-object manipulation, and contact-rich manipulation tasks [1,2,3,4]. In these applications, the arm must operate with limited onboard power and actuator torque, while large distal inertia reduces dynamic performance and payload margin. Centrally driven and tendon- or linkage-transmitted arms therefore offer a promising route to reduce moving inertia, but their advantages can only be fully exploited if the planner selects postures that make the arm easier to support and drive.

Redundancy resolution remains a central problem in humanoid arm motion planning. A recent review classifies redundancy-resolution methods into analytical, numerical, optimization-based, and learning-based families and highlights recurring trade-offs among computational efficiency, constraint handling, and solution transparency [5]. Position-level and numerical inverse-kinematics methods can accommodate general kinematic structures, but their iterative nature does not directly expose the complete feasible interval of a human-arm-like redundancy parameter [6,7,8]. Differentiable-manifold and cyclic-coordinate formulations provide mathematically general descriptions of self-motion, although they introduce a higher-dimensional representation and additional numerical processing for path-wise constraint evaluation [9,10,11]. Redundancy-parameter and specialized analytical formulations can compactly represent self-motion for particular manipulator families, but most primarily address kinematic feasibility rather than posture-dependent actuator loading [12,13]. Manipulability optimization improves dexterity or velocity/force transmission locally, yet a high manipulability index does not by itself guarantee that a specified payload can be supported within individual actuator-torque limits [14,15]. Model-free and recurrent-neural formulations can avoid conventional explicit inverse-kinematics inversion, but feasibility is enforced through online control or iterative dynamics, and neither formulation directly exposes an actuator-based static payload-margin interval [16,17]. Energy-aware redundancy resolution can instead embeds minimum-consumption objectives in the online solver, as demonstrated by a fuzzy-control-aided zeroing neural network, but this optimization does not provide an explicit payload-feasible arm-angle interval [18]. For humanoid object pick-and-place, task-priority redundancy resolution has been used to pursue minimum energy while maintaining dynamic balance, but this work focuses on whole-body task coordination rather than an actuator-wise static payload margin [19].

Constrained planners can incorporate joint limits, self-collision, obstacle avoidance, dynamic feasibility, task constraints, and actuator bounds [20,21,22,23]. Torque-bounded task-space admittance control directly enforces actuator limits during interaction, but addresses feedback-control behavior rather than construction of a static payload-feasible redundancy interval [24]. Predictive inverse-kinematics and trajectory-scaling methods can enforce motion and feasibility constraints along a prescribed path [25,26,27,28]. Their generality is valuable, but repeated collision checking or online optimization can be computationally demanding, and actuator feasibility is commonly imposed as a trajectory-level constraint rather than expressed as an interpretable posture interval. Learning-based payload-manipulation methods can generate dynamics-compliant trajectories for super-nominal payloads, although they rely on learned trajectory distributions and do not yield an analytical posture-wise payload margin [29]. Robust path-velocity control can also account for torque limits under uncertainty, but treats feasibility through trajectory timing and feedback rather than through a load-capacity interval in the redundancy coordinate [30]. Minimum-time, uncertainty-aware, reinforcement-learning, and task/input-constrained planners face related trade-offs between generality, computational cost, and model or data dependence [31,32,33,34,35]. Torque- and trajectory-aware methods optimize actuator loading, smoothness, or related dynamic objectives, but they generally do not convert a prescribed endpoint load into an explicit posture-wise feasible interval in the redundancy coordinate [36,37,38,39].

The unresolved gap is therefore a transparent and computationally compact method that converts both kinematic feasibility and payload-support capability into the same redundancy coordinate for a centrally driven humanoid arm. For a fixed wrist-center target, different arm angles can produce markedly different joint-torque distributions even though all of them satisfy the Cartesian task. The proposed study addresses this gap by projecting joint limits, singularities, and a static actuator-torque-based load-capacity model into the one-dimensional arm-angle domain. Their intersection gives an explicit posture interval from which a continuous trajectory can be selected without repeatedly optimizing in the full joint space.

This study develops a posture-planning method for a four-degree-of-freedom centrally driven humanoid robotic arm. The arm uses a shoulder–elbow arrangement that is kinematically redundant for positioning the wrist center. Previous work introduced the arm architecture and an inverse-kinematics framework that already incorporated geometric and load-capacity constraints [40]. The present study extends that posture-level framework to continuous path-level arm-angle planning through explicit feasible-interval intersection and a trajectory-selection objective. The main contributions are as follows:

- A closed-form arm-angle formulation maps each Cartesian target and inverse-kinematics branch to a one-dimensional geometric feasible set that explicitly accounts for joint limits and singularities.
- A posture-dependent static load-capacity constraint combines gravity torque, payload mapping, and individual actuator limits, allowing mechanical feasibility to be evaluated directly in the arm-angle domain.
- A continuous planning procedure operates on the intersection of the geometric and mechanical feasible sets, thereby selecting payload-supporting postures without changing the prescribed Cartesian path.

The rest of this paper is organized as follows. Section 2 introduces the arm platform and kinematic model. Section 3 presents the arm-angle feasible-domain formulation and planning method. Section 4 reports simulation and prototype validation. Section 5 discusses the implications and limitations, and Section 6 concludes the paper.

## 2. Robotic Arm Platform and Kinematic Model

### 2.1. Centrally Driven Humanoid Arm

The experimental platform is a lightweight humanoid robotic arm whose actuators are concentrated near the shoulder. The design is inspired by the proximal antagonistic actuation of the human elbow, but rigid linkage transmissions are used instead of compliant artificial muscles. Most structural components are machined from 7075 aluminum alloy, while the S-shaped inner connecting rod is stainless steel to provide higher strength in the compact transmission path. This paper does not repeat the complete mechanical design reported in [40]; only the parameters required for planning and validation are summarized here.

The arm has four positioning degrees of freedom in a “2–2” arrangement. The shoulder provides two rotational degrees of freedom, and the elbow provides two rotational degrees of freedom. The wrist module is not considered in this study because the planning target is the wrist-center position. The two elbow actuators are placed near the proximal end of the upper arm rather than at the distal elbow. Their motion is transmitted to the elbow plate and forearm through paired rigid four-bar mechanisms. The outer mechanism drives elbow abduction–adduction with two symmetric planar four-bar linkages, and the inner mechanism drives forearm flexion–extension through a spatial four-bar linkage. This arrangement follows the antagonistic actuation principle of the human arm while using rigid transmission components for predictable modeling and control. An S-shaped inner connecting rod is used to avoid interference with the internal elbow motor and to enlarge the practical motion range.

**Figure 1:**
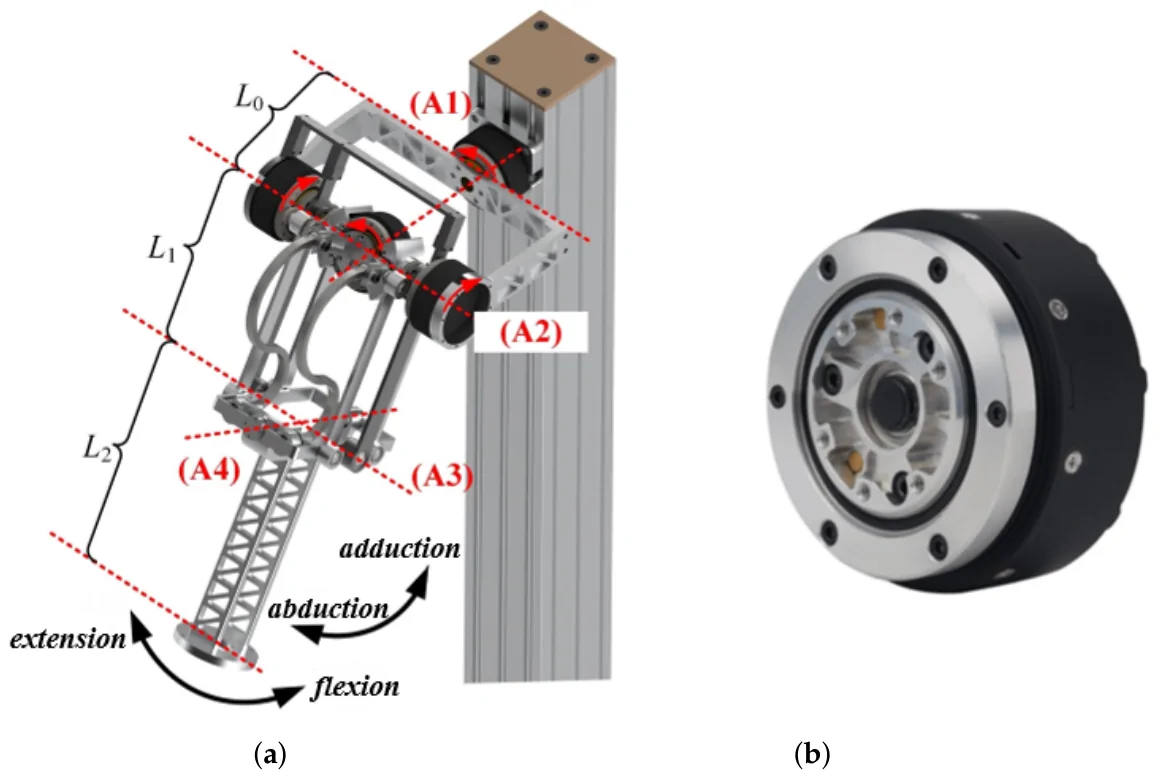
Three-dimensional structure of the centrally driven humanoid robotic arm and the selected joint drive motor: (**a**) arm structure, where A1–A4 denote the four actuated joint axes from the shoulder side to the elbow side; (**b**) joint actuator used in the arm.

The joint actuator is a compact Unitree A1 motor with a mass of 0.605 kg, a peak output torque of 33.5 Nm, a maximum speed of 21 rad/s, and a rated DC supply range of 20–40 V. Each actuator contains an absolute position encoder; the motor drive returns joint position and angular velocity for state monitoring and closed-loop joint control. The arm structure and actuator are shown in Figure 1, and the basic physical parameters are listed in Table 1. The arm mass is approximately 6.1 kg and the arm span is 0.663 m. The previously reported theoretical static payload of 5.9 kg applies to the specified reference posture under the ideal peak-torque model; it is not a workspace-wide or continuous-operation guarantee. Compared with an equivalent serial arrangement, the centralized layout reduces the maximum inertia of the elbow drive unit about the shoulder axes by 76.64% and 64.84%, respectively.

The scope of this study is wrist-center position planning under joint-range, singularity, and static load-capacity constraints. Collision detection and obstacle avoidance are not implemented on the present prototype: the laboratory trajectories are checked in advance to be free of environmental and self-collision, and the arm has no dedicated contact or proximity sensor used by the planner. Likewise, force control, grasp planning, and hand dexterity are outside the scope because no wrist/hand module is included. Positioning performance is evaluated experimentally in Section 4. A deployable manipulation system would need to combine the proposed arm-angle feasible set with exteroceptive obstacle sensing, collision monitoring based on force/torque or motor-current residuals, and a task-level hand controller.

### 2.2. Forward Kinematics and Singularity

**Figure 2:**
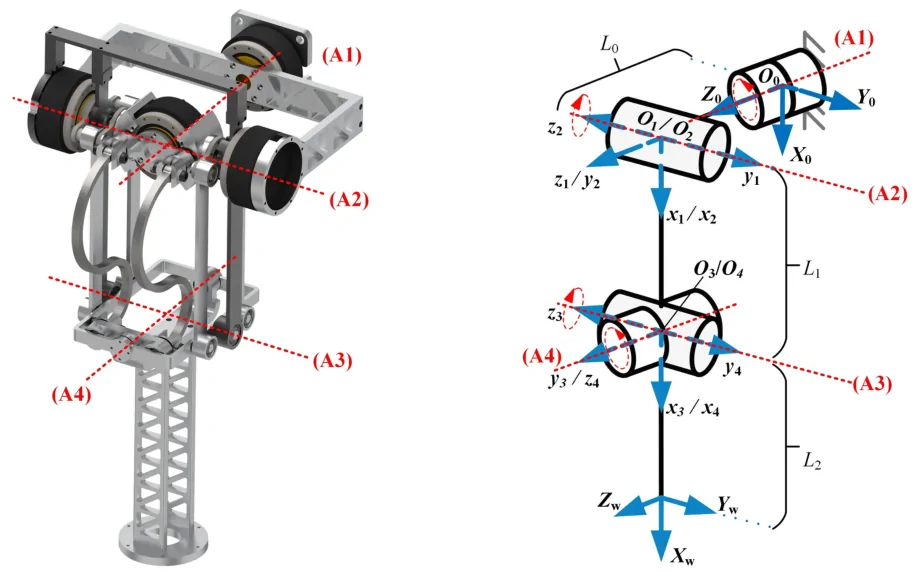
Kinematic model and coordinate-system definition of the robotic arm used for forward kinematics, inverse kinematics, and Jacobian analysis; A1–A4 denote the four actuated joint axes from the shoulder side to the elbow side.

The arm is modeled with modified Denavit–Hartenberg parameters, as shown in Figure 2. Let the joint vector be.

(1) $$\theta ={\left[\theta_{1},\theta_{2},\theta_{3},\theta_{4}\right]}^{T}$$

where $\theta_{1}$ and $\theta_{2}$ describe the shoulder-side rotations and $\theta_{3}$ and $\theta_{4}$ describe the elbow-side rotations.

The homogeneous transformation from the base frame to the wrist-center frame is

(2) TW0(θ)=T10T21T32T43TW4

The wrist-center position is denoted as

(3) $$p_{W}={\left[x_{W},y_{W},z_{W}\right]}^{T}=f\left(\theta \right)$$

The analytical forward model was checked against a Robotics Toolbox model by Monte Carlo sampling. With 10,000 randomly generated joint configurations, the coordinate error between the two models was on the order of numerical precision, confirming the correctness of the kinematic model. The same sampling strategy was used to visualize the reachable workspace of the wrist center. The workspace result provides the geometric boundary for selecting feasible Cartesian paths before arm-angle planning is performed.

For position-level planning, the relevant Jacobian is the translational Jacobian $J_{v}\left(\theta \right)$:

(4) $${\dot{p}}_{W}=J_{v}\left(\theta \right)\dot{\theta }$$

The singularity analysis shows two principal singular configurations. The first is a shoulder singularity, where one shoulder degree of freedom becomes ineffective. The second is an elbow singularity, where the upper arm and forearm become collinear. A normalized manipulability index was also used in the workspace evaluation to assess local motion dexterity. The index is low near the workspace boundary and in part of the upper workspace, while the middle workspace generally provides better motion performance. These singularity and dexterity observations are used later to exclude unsafe arm-angle neighborhoods and to interpret why some target paths are more favorable than others.

## 3. Load-Capacity-Constrained Arm-Angle Planning

### 3.1. Arm-Angle Description

**Figure 3:**
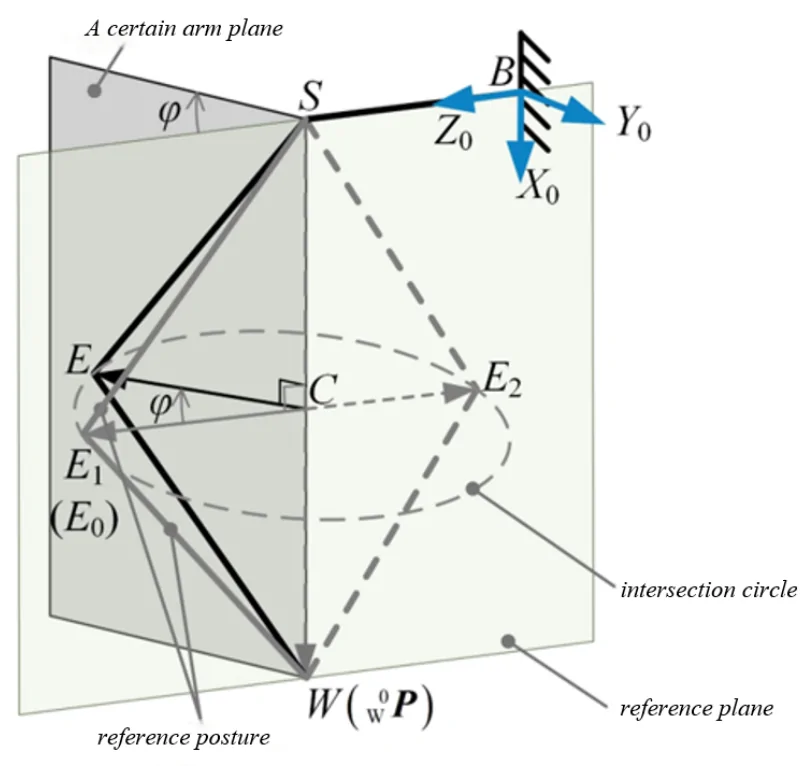
Arm-angle posture description. The elbow moves on the intersection circle while the wrist-center point is fixed; the arm angle φ measures the rotation from the reference posture to the selected arm plane.

Let *S*, *E*, and *W* denote the shoulder center, elbow center, and wrist center, respectively. For a given wrist-center point *W*, the distances $∥SE∥=L_{1}$ and $∥EW∥=L_{2}$ are fixed. Therefore, the possible elbow positions lie on the intersection circle of two spheres centered at *S* and *W*. This intersection circle represents the one-dimensional redundancy of the arm posture, as illustrated in Figure 3.

A reference elbow point $E_{0}$ is defined on the reference plane determined by the base–shoulder direction and the shoulder–wrist direction. For special cases in which this plane is not unique, a fixed vertical reference plane is selected. Any feasible elbow point *E* can then be obtained by rotating $E_{0}$ around the shoulder–wrist axis. The rotation angle is the arm angle φ:

(5) $$e\left(\varphi \right)=c+R\left(u,\varphi \right)\left(e_{0}-c\right)$$

where e, $e_{0}$, and c are the position vectors of *E*, $E_{0}$, and the circle center *C*, respectively; u is the unit vector from *S* to *W*; and R(u,φ) is computed by Rodrigues’ formula:

(6) $$R\left(u,\varphi \right)=I\cos\varphi +\left(1-\cos\varphi \right)uu^{T}+{\left[u\right]}_{\times }\sin\varphi$$

Thus, for a fixed target point, the redundant posture is converted into a scalar variable φ.

### 3.2. Closed-Form Inverse Kinematics

For each target point $p_{W}$ and arm angle φ, Equation (5) determines the elbow point. The joint configuration is then solved from the geometric relationship among *S*, *E*, and *W* and from the forward kinematic equations. A global configuration parameter $GC_{2}$ is used to distinguish the two inverse-kinematics branches associated with $\theta_{2}$:

(7) $$GC_{2}=\left\{\begin{array}{cc} +1, & \theta_{2}\geq \frac{\pi }{2},\\ -1, & \theta_{2}<\frac{\pi }{2}. \end{array}\right.$$

Following the branch convention used in the source inverse-kinematics derivation, $GC_{2}=1$ denotes $\theta_{2}\geq \pi /2$, whereas $GC_{2}=-1$ denotes $\theta_{2}<\pi /2$. The parameter fixes the discrete shoulder/elbow assembly mode before the continuous arm angle is varied. The inverse-kinematics mapping can therefore be written as

(8) $$\theta =\Theta (p_{W},\varphi ,GC_{2})$$

This closed-form mapping makes it possible to search and optimize in the one-dimensional arm-angle domain instead of the full joint space.

### 3.3. Geometric Feasible Set

The joint limits define the first arm-angle feasible set. For the *i*th joint, the limit constraint is

(9) $$\theta_{i}^{\min}\leq \Theta_{i}\left(p_{W},\varphi ,GC_{2}\right)\leq \theta_{i}^{\max}$$

The corresponding arm-angle interval set is denoted by $\Phi_{i}\left(p_{W},GC_{2}\right)$. The joint-limit feasible set is the intersection of the interval sets for all joints:

(10) $$\Phi_{J}\left(p_{W},GC_{2}\right)=\overset{4}{\underset{i=1}{⋂}}\Phi_{i}\left(p_{W},GC_{2}\right)$$

The singularity constraint further removes unsafe arm angles. The elbow singularity occurs when $∥\vec{SW}∥=L_{1}+L_{2}$; all arm angles are singular at that target. The shoulder singularity at φ=0 applies only when the reference elbow satisfies $x_{E_{0}}=0$ and $y_{E_{0}}=0$. The geometric feasible set is therefore

(11) $$\Phi_{G}\left(p_{W},GC_{2}\right)=\left\{\begin{array}{cc} ⌀, & ∥\vec{SW}∥=L_{1}+L_{2},\\ \Phi_{J}\left(p_{W},GC_{2}\right)\setminus U\left(0,\delta \right), & x_{E_{0}}=0\;\wedge \;y_{E_{0}}=0,\\ \Phi_{J}\left(p_{W},GC_{2}\right), & \mathrm{otherwise}. \end{array}\right.$$

In numerical implementation, the two zero-coordinate tests require a tolerance selected consistently with the kinematic solver.

### 3.4. Load-Capacity Model

The mechanical constraint is based on static load capacity. The actuator torque is divided into two parts: the torque required to balance the arm’s own weight and the torque required to balance the external payload. In the static model, stainless steel is used for the inner connecting rods and aluminum alloy is used for the remaining main components. An Adams View 2020 virtual prototype was also created from the SOLIDWORKS 2025 model to check the static load analysis and to support later trajectory simulations. Reducer friction, seal friction, and transmission hysteresis are not included in the analytical capacity equation because their direction- and speed-dependent parameters were not independently identified. Consequently, the model predicts posture-dependent rigid-body torque demand rather than the complete electrical input. In the prototype tests, these losses are not numerically compensated; they are inherently present in the measured actuator response and energy consumption, which is one reason that experimental savings are smaller than the ideal simulation prediction.

**Figure 4:**
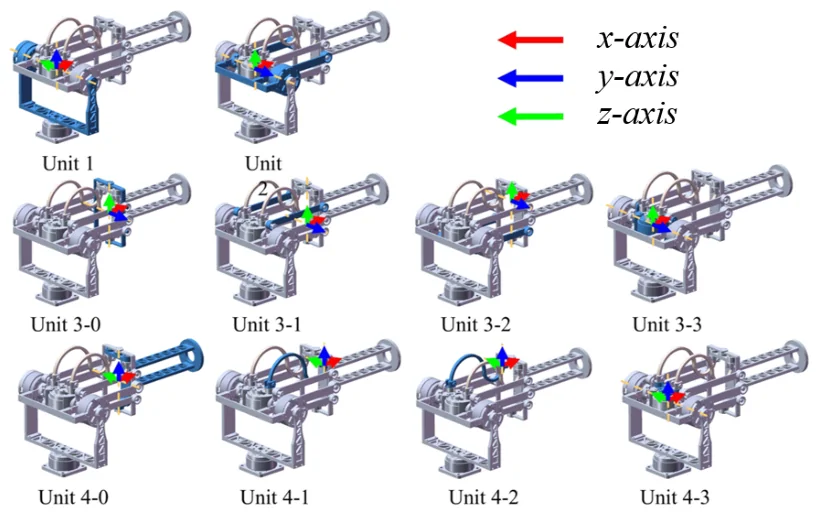
Mass-unit division and coordinate-system arrangement used for the Lagrangian gravity-torque model.

The gravity torque is obtained from a Lagrangian formulation. The arm is divided into rigid mass units, as shown in Figure 4.

The total potential energy is written as

(12) $$V\left(\theta \right)=\sum_{k}m_{k}gh_{k}\left(\theta \right)$$

where $m_{k}$ is the mass of the *k*th unit and $h_{k}$ is the height of its center of mass in the gravity direction. Under static conditions, the gravity torque is

(13) $$\tau_{g}\left(\theta \right)=\frac{\partial V(\theta )}{\partial \theta }$$

The main mass units used in the model are listed in Table 2.

For an external Cartesian force F0 expressed in the base frame and applied at the wrist center, the equivalent joint torque is obtained by the principle of virtual work:

(14) τF=JvT(θ)F0

For the downward payload used in the source coordinate convention, f^0=[1,0,0]T. Let F≥0 be the force magnitude in newtons, so F0=Ff^0. The total static torque is

(15) τ(θ,F)=τg(θ)+FJvT(θ)f^0

The source derivation used a componentwise limiting-load ratio under a fixed force direction. Here, it is reformulated as a corrected two-sided torque-feasibility condition. Given symmetric actuator limits $|\tau_{i}|\leq \tau_{i}^{\max}$, define ai(θ)=[JvT(θ)f^0]i. The configuration-level static load capacity is

(16) $$F_{\max}\left(\theta \right)=\sup\left\{F\in R_{\geq 0}:-\tau_{i}^{\max}\leq \tau_{g,i}\left(\theta \right)+Fa_{i}\left(\theta \right)\leq \tau_{i}^{\max},\;i=1,\ldots ,4\right\}$$

For implementation, when $a_{i}\neq 0$, actuator *i* defines the interval between $\left(-\tau_{i}^{\max}-\tau_{g,i}\right)/a_{i}$ and $\left(\tau_{i}^{\max}-\tau_{g,i}\right)/a_{i}$; these intervals are intersected with F≥0. When $a_{i}=0$, the actuator imposes no payload-dependent bound if $|\tau_{g,i}|\leq \tau_{i}^{\max}$ and makes the configuration infeasible otherwise. This construction covers cases in which gravity and payload torques act in either the same or opposite directions. Combining Equations (8) and (16), the load capacity becomes a function of the target point and arm angle:

(17) $$F_{\max}=F_{\max}\left(p_{W},\varphi ,GC_{2}\right)$$

For a task requiring a minimum payload $F_{\mathrm{req}}$, the mechanical feasible set is

(18) $$\Phi_{M}\left(p_{W},GC_{2}\right)=\left\{\varphi |F_{\max}\left(p_{W},\varphi ,GC_{2}\right)\geq F_{\mathrm{req}}\right\}$$

The final feasible set is the intersection

(19) $$\Phi_{\mathrm{GM}}=\Phi_{G}\cap \Phi_{M}$$

### 3.5. Path Planning Procedure

For a Cartesian path discretized into *N* target points ${\left\{p_{W}\left(n\right)\right\}}_{n=1}^{N}$, the planner performs the following steps:

1. Compute the geometric feasible set $\Phi_{G}\left(n\right)$ for each point.
2. Compute the load-capacity feasible set $\Phi_{M}\left(n\right)$ under the specified payload requirement.
3. Intersect the two sets to obtain $\Phi_{\mathrm{GM}}\left(n\right)$.
4. Select a continuous and smooth arm-angle sequence {φ(n)} inside $\Phi_{\mathrm{GM}}\left(n\right)$.
5. Use the closed-form inverse kinematics to obtain the joint path and then assign time to obtain the joint trajectory.

**Figure 5:**
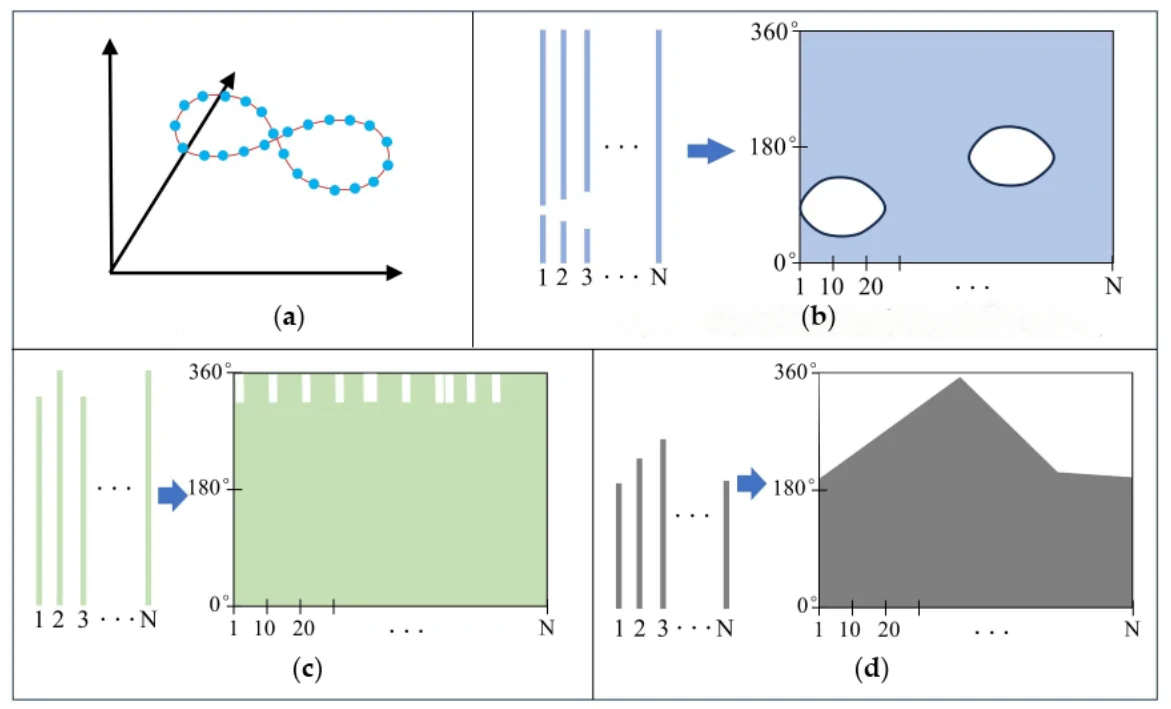
Target-path discretization and arm-angle feasible-range acquisition: (**a**) target-path discretization; (**b**) arm-angle feasible range under the *i*th joint-range constraint; (**c**) arm-angle feasible range under singularity constraints; (**d**) arm-angle feasible range under load-capacity constraints.

Figure 5 summarizes the path discretization and the construction of the arm-angle feasible range from joint-range, singularity, and load-capacity constraints.

The source planning procedure specifies sequential selection of a continuous and smooth arm-angle curve but does not provide an explicit objective function. Equation (20) is an algorithmic formalization introduced in this manuscript rather than a transcription of the source derivation. Define the periodic angular distance as d(α,β)=|atan2(sin(α−β),cos(α−β))|. At the *n*th point, after selecting a nonzero-width feasible interval, the arm angle minimizes the dimensionless cost

(20) $$\varphi \left(n\right)=\arg\min_{\varphi \in \Phi_{\mathrm{GM}}\left(n\right)}\left[c{\left(\frac{d(\varphi ,\varphi_{c}\left(n\right))}{\Delta \varphi (n)}\right)}^{2}+\left(1-c\right){\left(\frac{d(\varphi ,\varphi (n-1))}{\pi }\right)}^{p}\right]$$

where $\varphi_{c}\left(n\right)$ and Δφ(n)>0 are the center and half-width of the selected feasible interval, φ(0) is the stated initial arm angle for the case, and π is the fixed normalization scale for the continuity term. Both cost terms are dimensionless, and *d* handles the 0/2π wrap-around. The weight c∈[0,1] balances distance from the interval center against continuity with the previous solution: increasing *c* moves the solution farther from constraint boundaries, whereas decreasing *c* emphasizes inter-point smoothness. The exponent p≥2 determines how strongly large arm-angle increments are penalized; a larger *p* suppresses abrupt changes more strongly. The values used in each case were selected empirically by starting from a continuity-dominant setting and increasing the centering penalty until the curve retained a visible margin from the feasible-set boundary. The same *c* and *p* were used for the geometric-only and geometric–mechanical planners within each case, so the comparison isolates the effect of the load-capacity constraint. A systematic sensitivity analysis of these parameters is left for future work. Because the optimization is one-dimensional, it is expected to require less computation than full joint-space trajectory optimization; runtime benchmarking on the target controller remains necessary before claiming real-time suitability.

**Figure 6:**
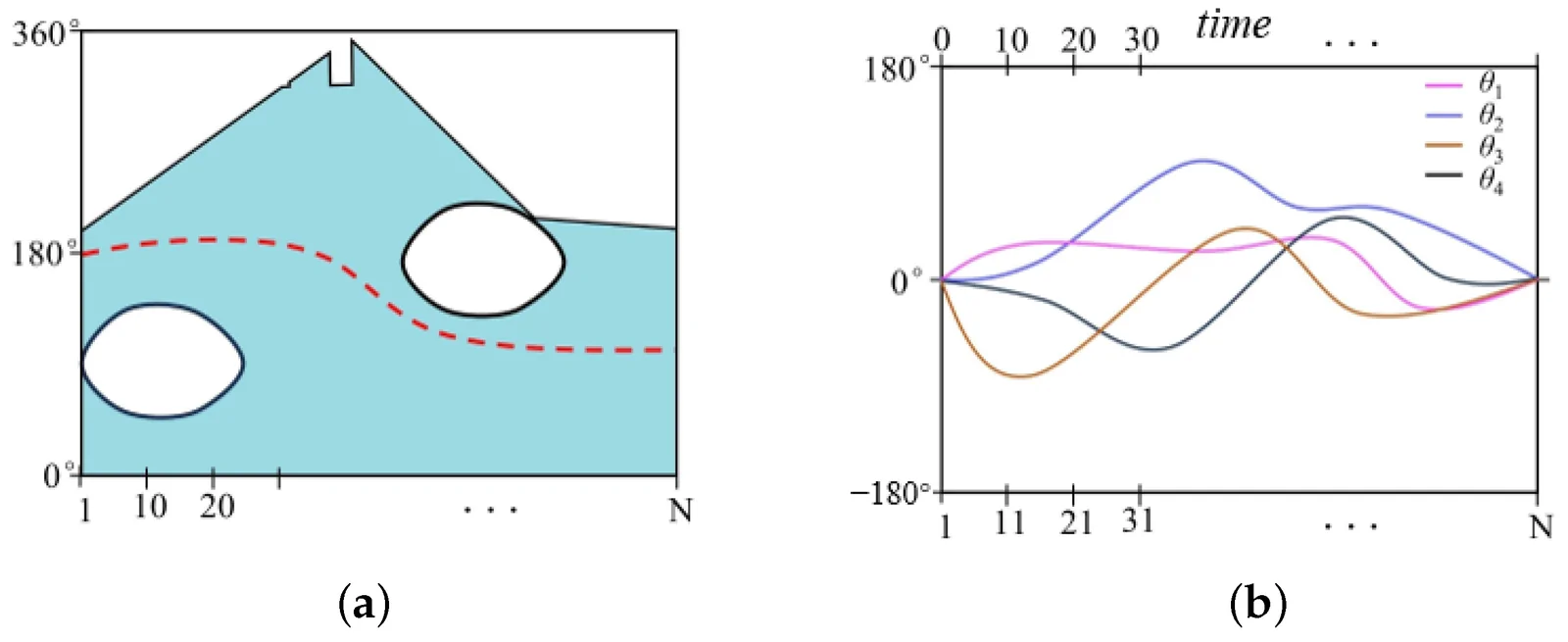
Posture and trajectory planning based on the global arm-angle feasible domain: (**a**) arm-angle curve in the global arm-angle feasible domain; (**b**) joint trajectories generated from the selected arm-angle curve. The red dashed line in (**a**) represents the selected arm-angle trajectory within the global feasible domain. Negative values in the plotted quantities use true minus signs.

The selected arm-angle curve is finally converted into joint trajectories through the closed-form inverse-kinematics mapping, as shown in Figure 6.

## 4. Simulation and Experimental Results

### 4.1. Validation of the Geometric Constraint

A representative target point was generated from θ=[60°,5°,40°,−50°]. The geometric feasible set was first computed analytically from the arm-angle constraint model. Then, the arm angle was discretized over [0°,360°) with a step of 0.5°, and all inverse-kinematics solutions were filtered by the joint limits. The feasible arm-angle intervals obtained by the two methods matched each other. The predefined configuration was also included in the feasible set, corresponding to an arm angle of approximately 148.95°. This comparison verifies the correctness of the arm-angle geometric constraint model.

### 4.2. Validation of the Load-Capacity Model

**Figure 7:**
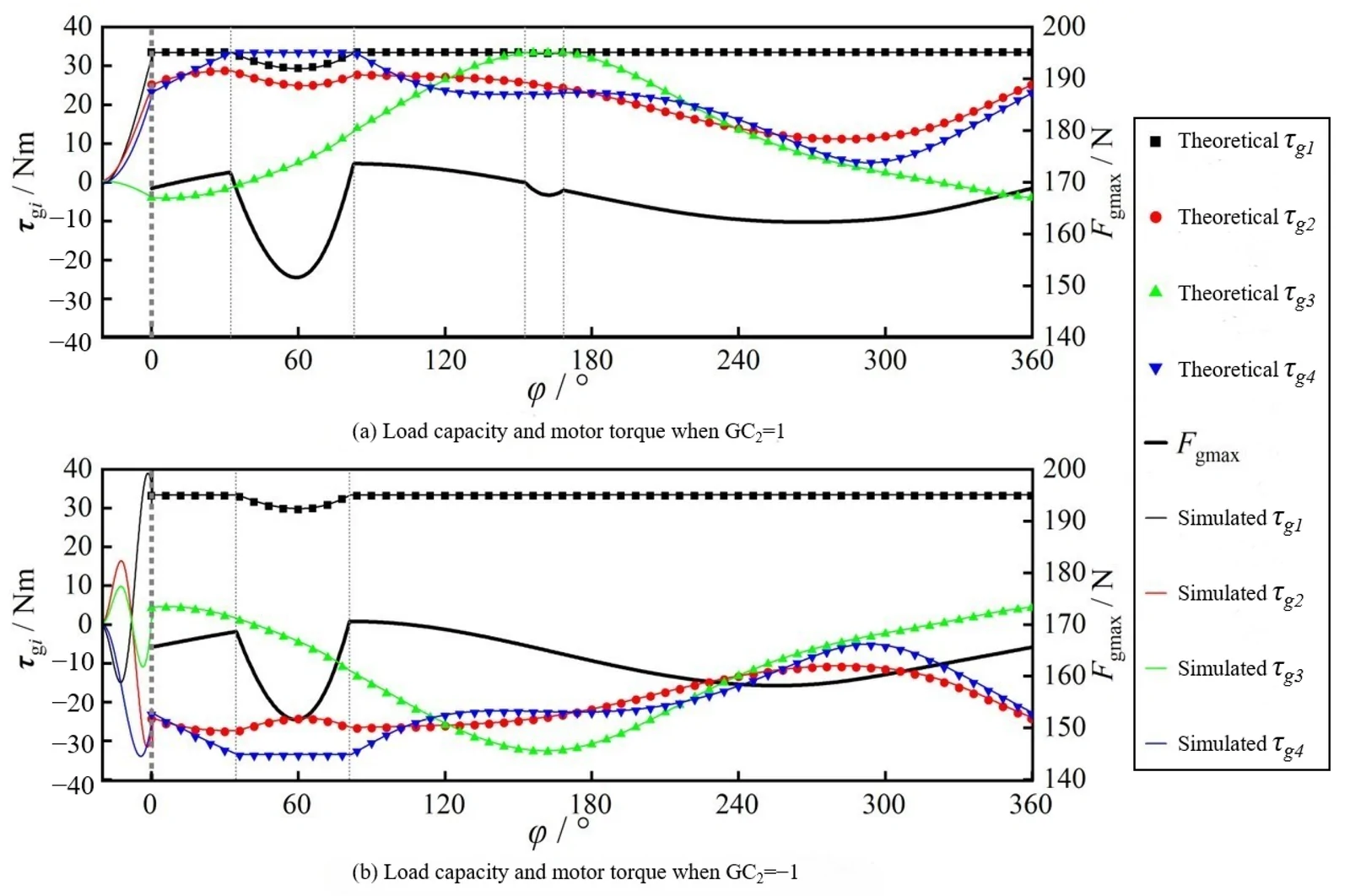
Comparison between the theoretical load-capacity model and Adams quasi-static simulation. Negative values in the plotted quantities use true minus signs. The limiting payload and joint torques vary with arm angle, and the model-predicted torques agree with the simulated torques.

For the same target point, the two inverse-kinematics branches were evaluated over the arm-angle range. The proposed load-capacity model produced the limiting load $F_{\max}\left(\varphi \right)$ and the corresponding joint torques. These arm-angle-dependent loads were then applied to an Adams virtual prototype under slow motion. As shown in Figure 7, the theoretical and simulated curves follow the same arm-angle-dependent trends and limiting-actuator transitions. The archived validation outputs do not contain the pointwise numerical data required to reconstruct a defensible maximum absolute error or root-mean-square error; therefore, this comparison is presented as a qualitative consistency check rather than a new quantitative accuracy claim.

This validation also shows why the mechanical constraint is necessary. For the same wrist-center position, the maximum bearable payload changes with the arm angle and with the inverse-kinematics branch. Therefore, selecting a mechanically favorable posture can increase the torque margin without modifying the end-effector path.

### 4.3. Simulation Case 1: Heavy-Load Spatial Path

The source study reports Adams output-power and energy metrics for both simulation cases, but the retained materials do not document the exact channel aggregation, sampling, numerical integration, or treatment of negative power. The values below are therefore reported as source simulation metrics rather than redefined as a newly reconstructed energy measure. Peak torque denotes the maximum reported output-torque magnitude over the evaluated trajectory.

The first simulation used a spatial double-twist path discretized into 144 points. The inverse-kinematics reconstruction error of the target path was on the order of $10^{-13}$ mm. A conservative planning torque limit of 8.375 Nm was imposed, equal to 25% of the motor’s reported 33.5 Nm peak output torque. This study-specific margin is not the actuator’s rated continuous torque or a measured protection threshold. The required downward load was 13 N, corresponding to an equivalent mass of approximately 1.33 kg under standard gravity; this conversion is not the measured payload limit. Instead, 13 N is a discriminating planning case under the conservative limit because it removes part of the geometric feasible domain while retaining a continuous solution for $GC_{2}=1$. Under this payload condition, the $GC_{2}=-1$ branch contained infeasible points, whereas the $GC_{2}=1$ branch provided a continuous feasible set.

**Figure 8:**
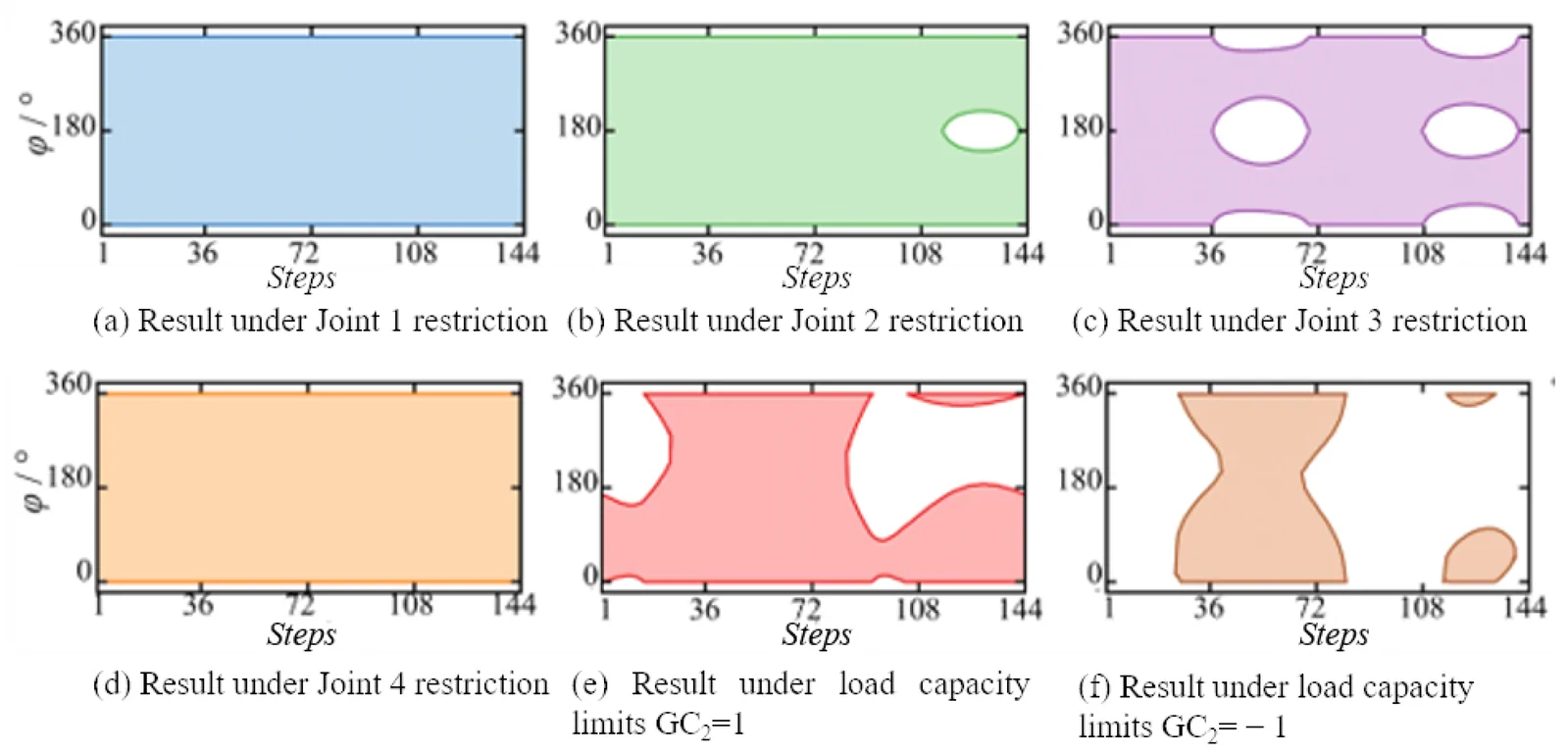
Arm-angle feasible domains of Simulation Case 1 under geometric and load-capacity constraints along the target path. Negative values in the plotted quantities use true minus signs.

**Figure 9:**
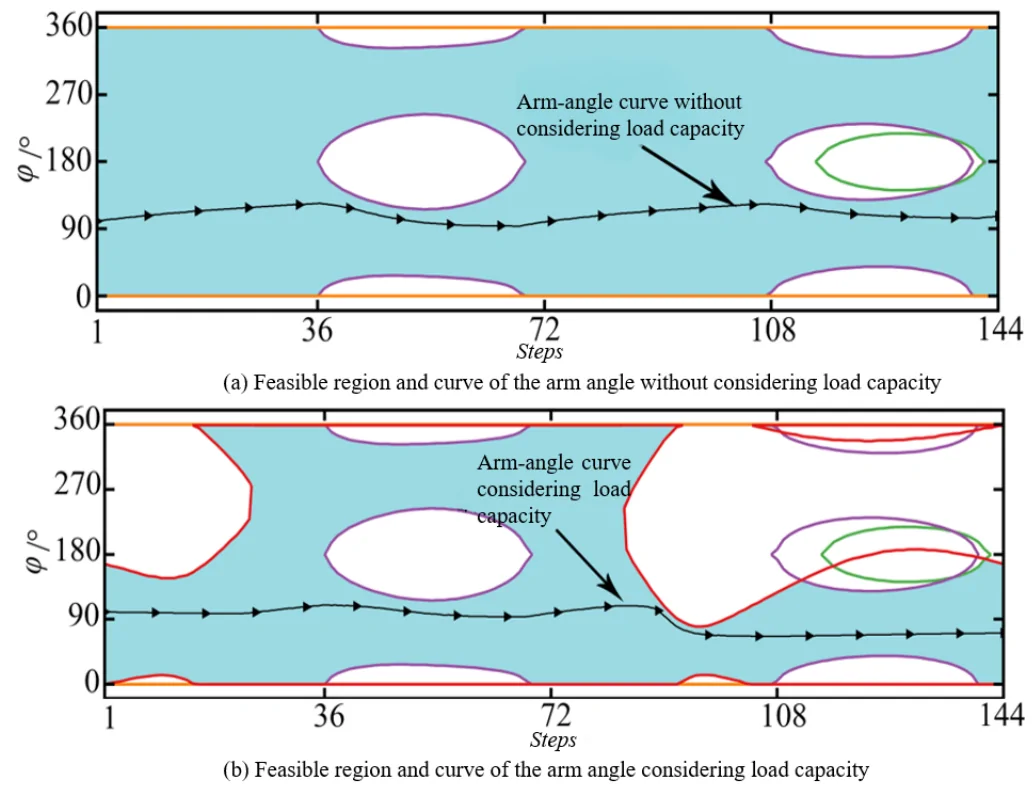
Global arm-angle feasible domain and selected arm-angle curve in Simulation Case 1. The lower panel includes the load-capacity constraint, which changes the feasible domain and the resulting posture trajectory.

Two arm-angle trajectories were compared. The first used only the geometric feasible set, and the second used the geometric–mechanical feasible set in Equation (19). Both trajectories were smoothed with the same optimization parameters, with an initial arm angle of 100°, c=0.3, and p=14. These values provide a continuity-dominant solution while penalizing occasional large inter-point changes and maintaining clearance from the feasible-set boundary. The joint paths were interpolated by Akima splines and executed in Adams over 58 s. Figure 8 and Figure 9 show how the load-capacity constraint changes the feasible domain and the selected arm-angle curve.

**Figure 10:**
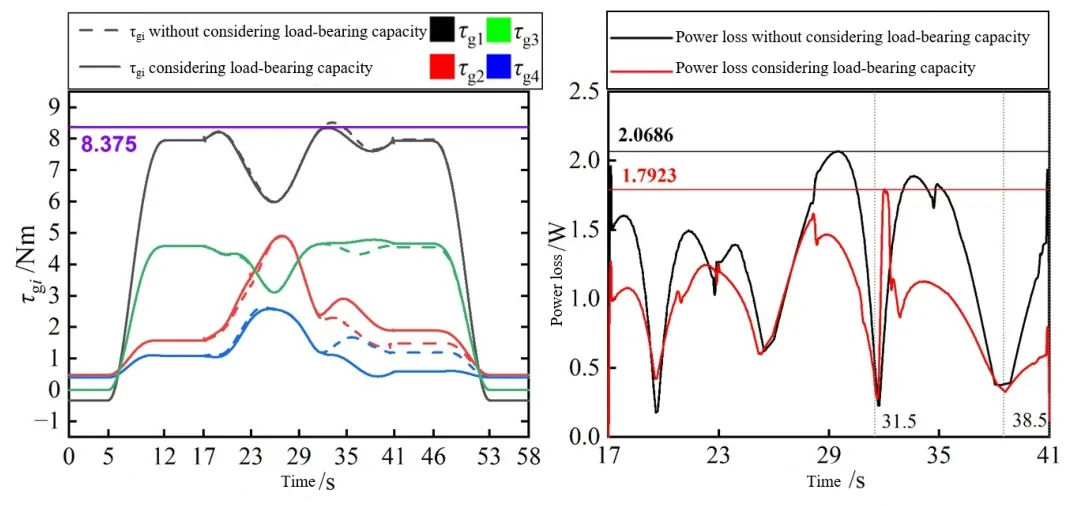
Adams trajectory-tracking simulation of Case 1. The load-capacity-constrained arm-angle trajectory keeps joint torques below the conservative planning torque limit and reduces peak positive mechanical power and integrated positive mechanical energy.

When only geometric constraints were considered, the maximum shoulder torque exceeded the conservative planning torque limit during the payload-tracking phase. In contrast, the proposed load-capacity-constrained trajectory kept all joint torques below that limit, as shown in Figure 10. The reported maximum output-power metric was 13.3% lower. The reported energy values decreased from 30.60 J to 22.95 J; recalculation from these source endpoints gives 7.65 J, or 25.0%, correcting the arithmetically inconsistent 33.3% stated in the source text.

### 4.4. Simulation Case 2: Large-Workspace Spiral Path

**Figure 11:**
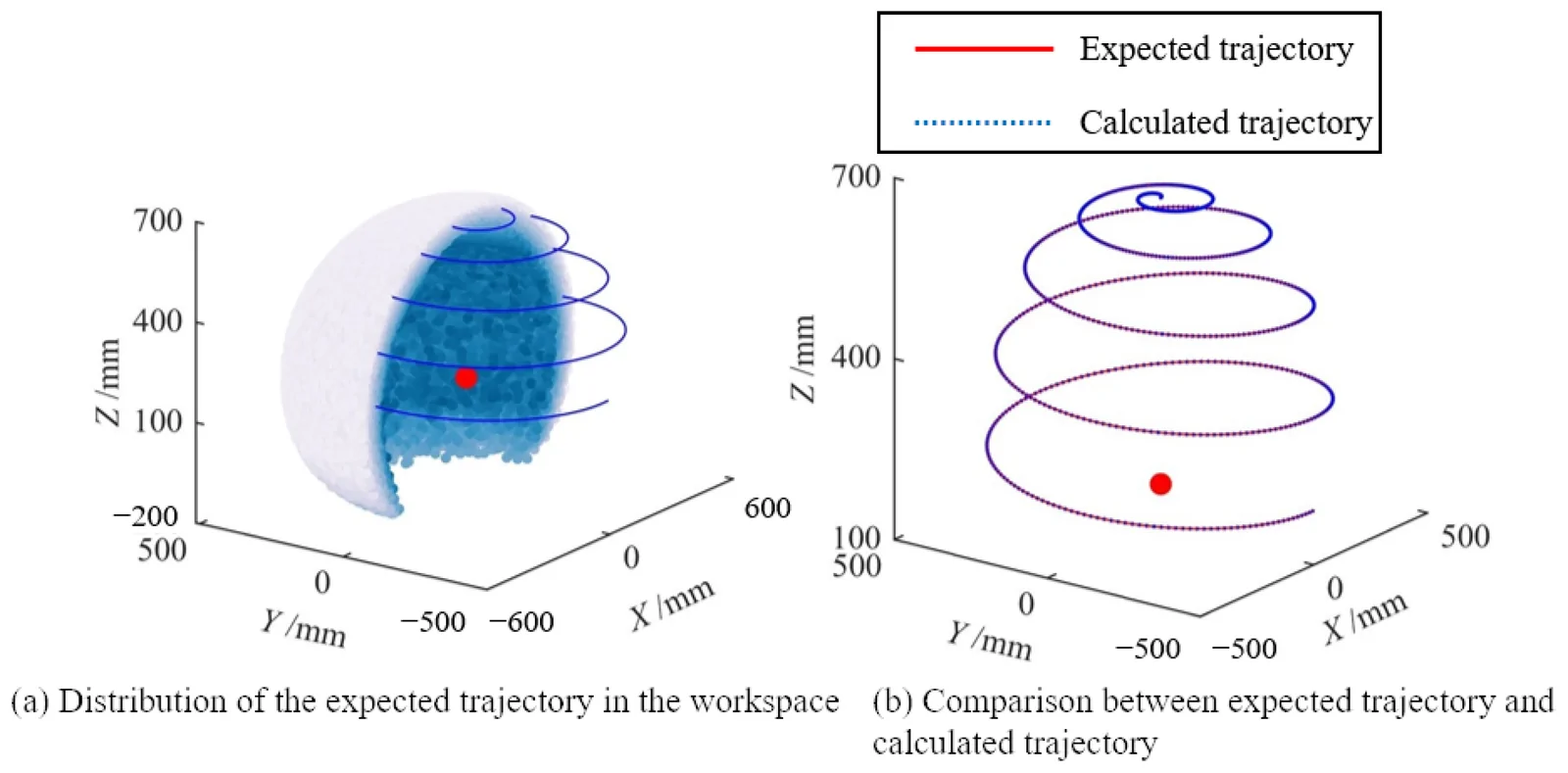
Large-workspace spiral path and forward/inverse-kinematics reconstruction in Simulation Case 2. Negative values in the plotted quantities use true minus signs.

The second simulation used a spiral trajectory that moved from the upper workspace downward while changing its radial distance from the shoulder. This path was discretized into 720 points and was selected to test the method over a broader workspace range. The same conservative planning torque limit of 8.375 Nm and the same load-capacity threshold of 13 N were used. The $GC_{2}=-1$ branch was discontinuous and therefore unsuitable, while $GC_{2}=1$ generated a valid feasible-domain sequence. Figure 11 shows the large-workspace path and the forward/inverse-kinematics reconstruction.

**Figure 12:**
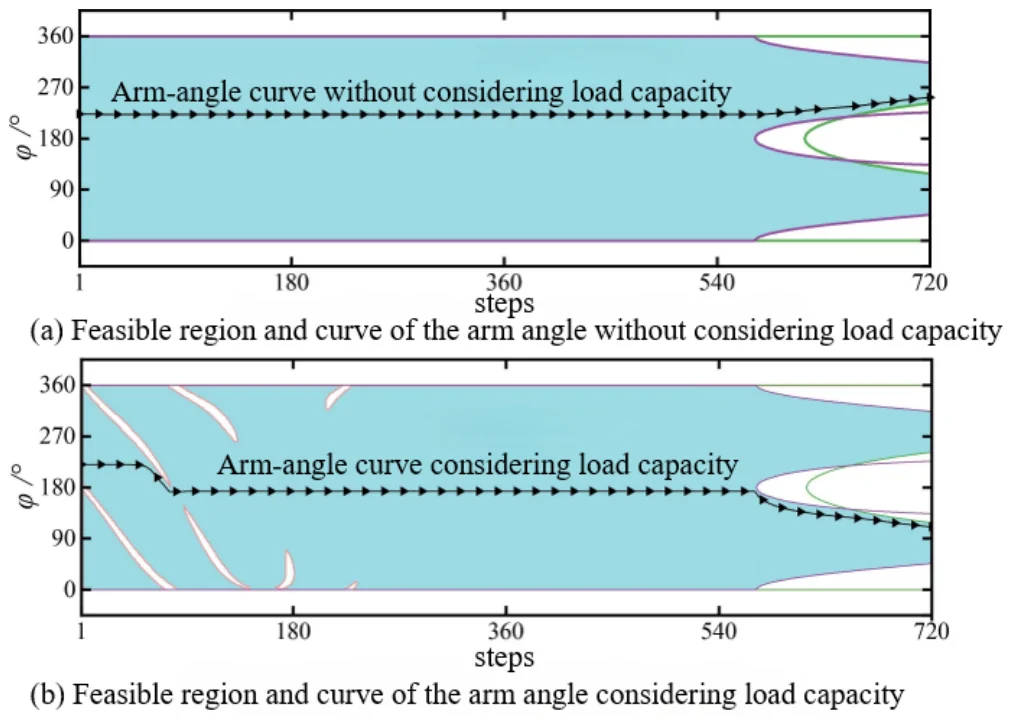
Global arm-angle feasible domain and selected arm-angle curve in Simulation Case 2.

**Figure 13:**
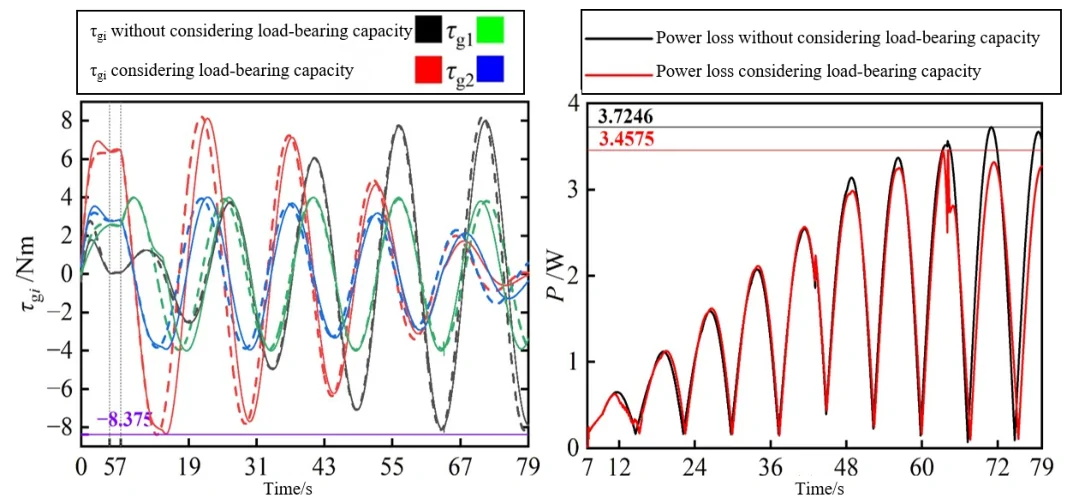
Trajectory-tracking simulation results of Simulation Case 2. The load-capacity-constrained trajectory reduces the maximum controller-recorded shoulder output torque and integrated positive mechanical energy over a broader workspace.

The initial arm angle was set to 230°, and the smoothing parameters were c=0.08 and p=25. The resulting feasible domain, arm-angle curve, and tracking results are shown in Figure 12 and Figure 13. The reported maximum output-torque values, corrected to Nm, decreased from 3.725 Nm to 3.456 Nm; recalculation from these endpoints gives 7.22%. The reported energy metric decreased from 119.88 J to 114.50 J, giving 4.49%. These percentages and the torque unit are corrections calculated from the source endpoint values. The result applies to the tested large-workspace path and does not establish performance throughout the workspace.

### 4.5. Prototype Experiments

**Figure 14:**
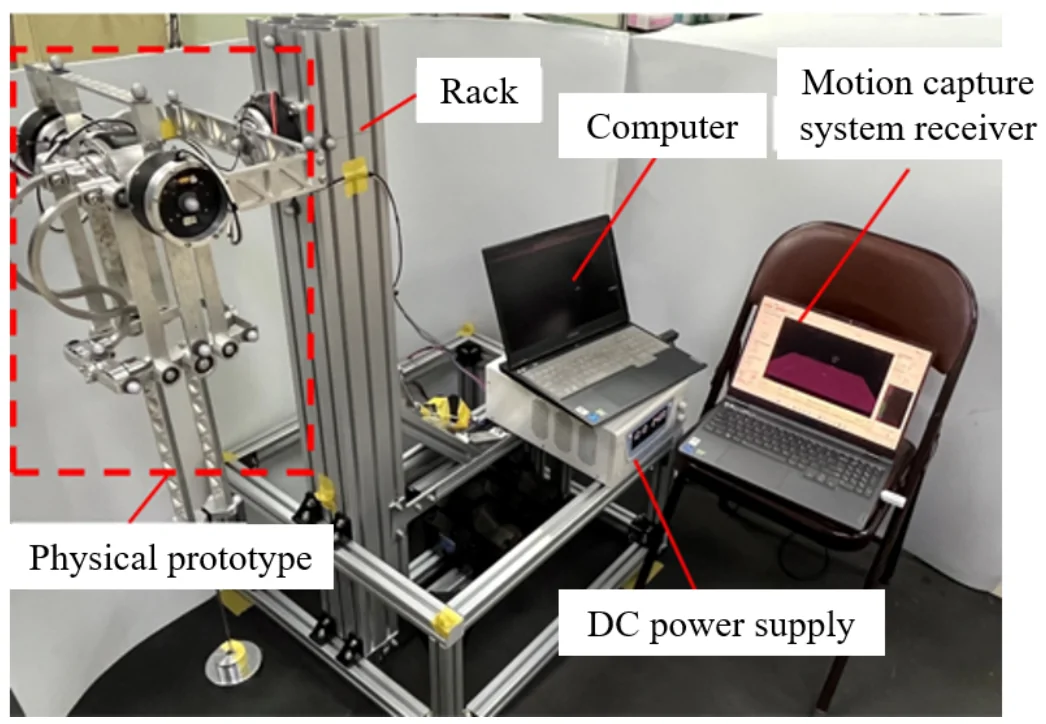
Physical prototype of the centrally driven humanoid robotic arm. The setup includes the arm, support frame, control computer, motion-capture receiver, and DC power supply.

**Figure 15:**
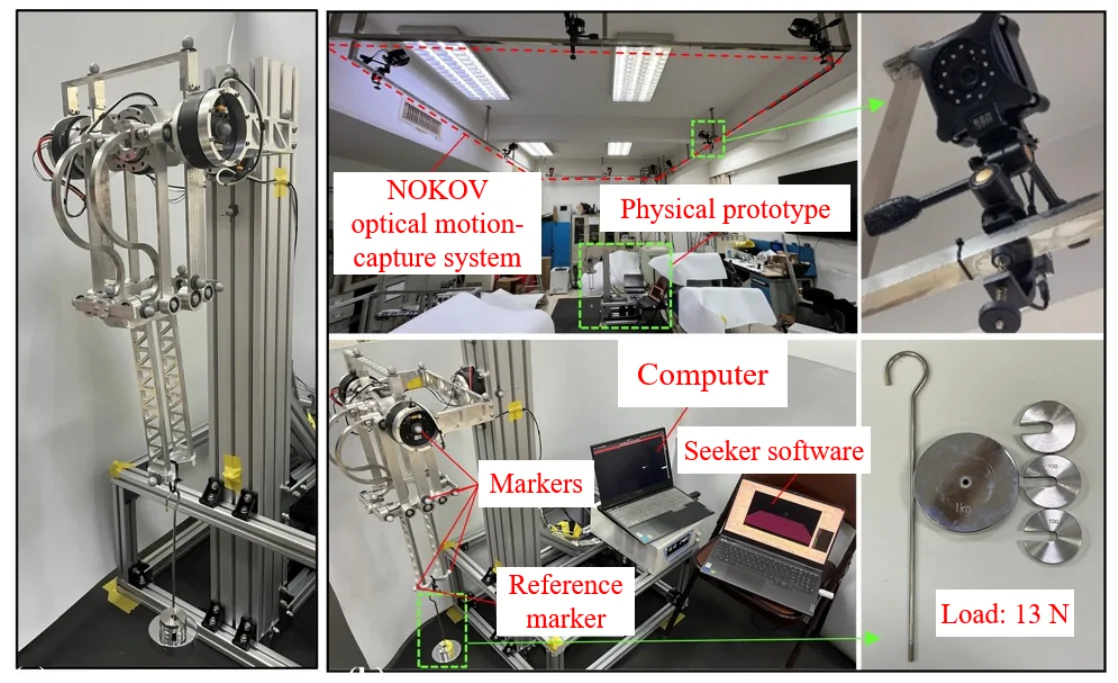
Prototype test platform with the optical motion-capture system and the robotic arm mounted on the support frame.

A physical prototype was built with the centrally driven arm, a joint motor control system, and an optical motion-capture system, as shown in Figure 14 and Figure 15. The controller used a feedforward-compensated PD structure, and the arm was operated through a client–server control framework. Joint angles were measured by the absolute position encoders integrated in the four Unitree A1 actuators. A NOKOV optical motion-capture system and Seeker software were used only as an independent external reference for endpoint-position evaluation. Before testing, the capture volume and world frame were calibrated, marker points were attached to the arm, and rigid bodies were defined for the relevant arm components. The captured data were post-processed by selecting the valid motion segment and transforming the measured points from the motion-capture world frame to the robotic-arm base frame. No collision-detection or obstacle-avoidance module was activated during these controlled tests; all commanded paths were verified to remain inside a cleared laboratory workspace. The archived prototype record labels the reported signals as controller-recorded shoulder torque and total energy. The shoulder peak is the maximum absolute recorded value over the payload-tracking segment. The original acquisition metadata needed to document torque calibration, sampling rate, filtering, electrical-power channels, and numerical integration were not preserved with the dataset used for this revision; consequently, the prototype torque and energy values are reported descriptively and are not assigned an uncertainty estimate or a reconstructed mean ± standard deviation.

**Figure 16:**
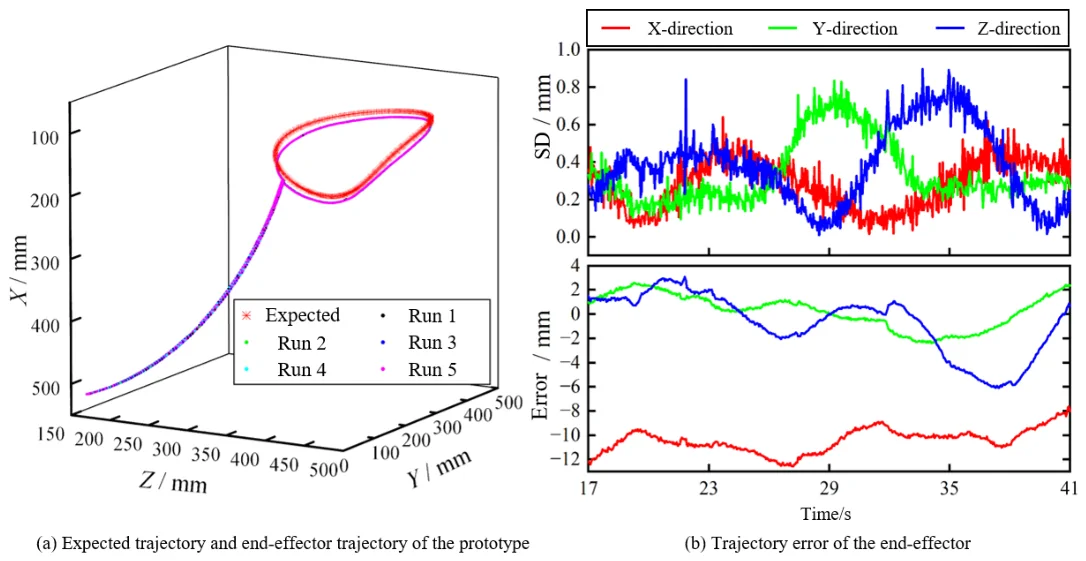
Endpoint trajectory tracking accuracy of the prototype. The repeated runs show high repeatability, while gravity-direction errors mainly come from structural compliance and assembly clearance.

The trajectory-tracking accuracy was evaluated by repeating the planned motion five times without payload. As shown in Figure 16, the repeatability was high: the standard deviation among the five runs was less than 1 mm in each coordinate direction. The mean tracking error was larger along the gravity-aligned axis, with an average value of approximately 10 mm, mainly due to link flexibility, assembly clearance, gearbox clearance, and self-weight deformation. In practical applications, this systematic component can be reduced by increasing link and joint stiffness, tightening machining and assembly tolerances, calibrating the loaded kinematic model, compensating gravity-dependent deflection, and closing the endpoint loop with external position sensing. These measures are engineering improvements rather than functions of the present arm-angle planner.

**Figure 17:**
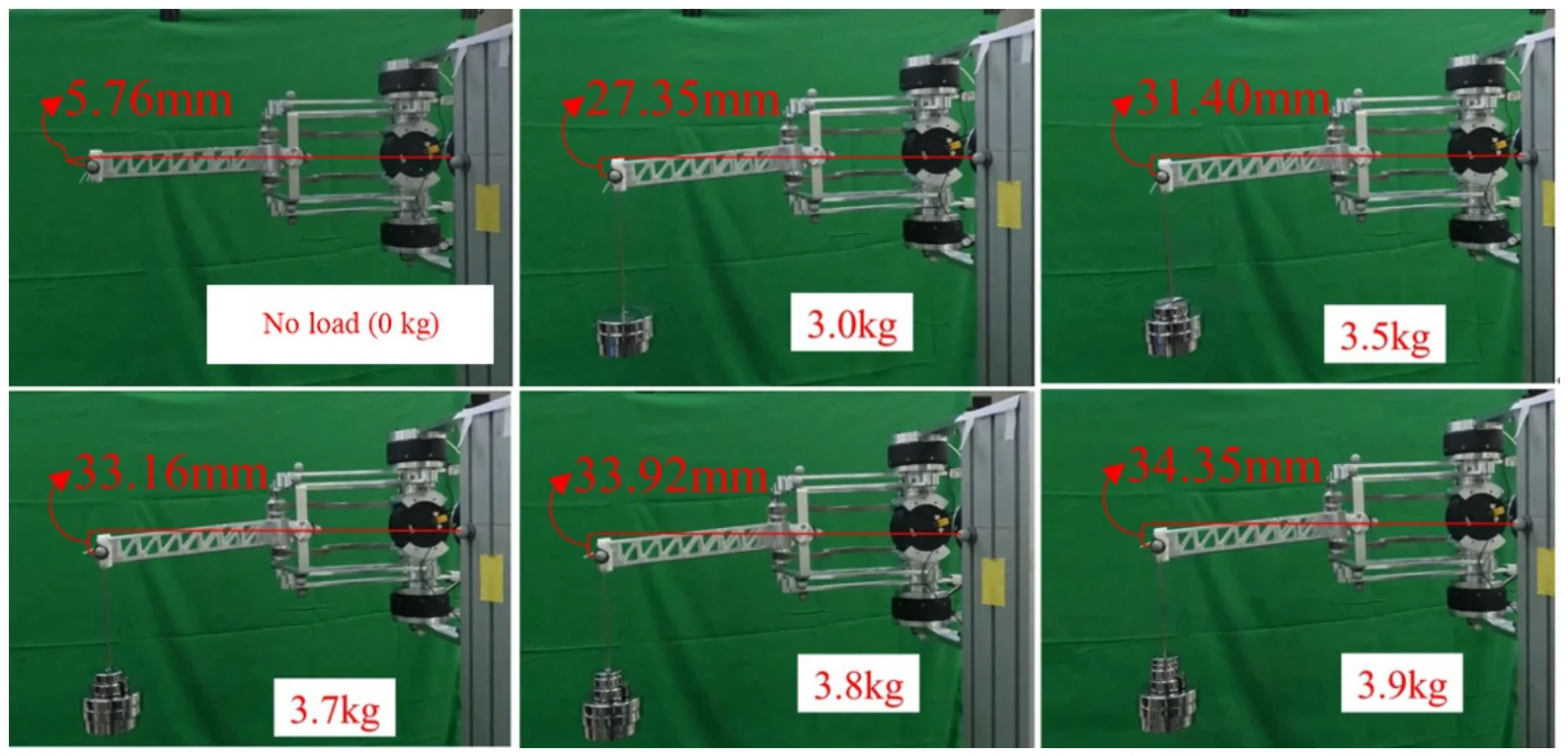
Endpoint loading experiment of the physical prototype used to evaluate practical static payload capability.

The static payload capability was tested with the arm in a forward horizontal posture, corresponding to a high shoulder-torque condition. The shoulder feedforward torque was set according to the nominal peak motor torque, and the endpoint payload was increased until the arm could no longer hold the load. To reduce heat accumulation in the joint motors, the test started from a 3 kg endpoint load and then used 100 g increments near the limiting condition. The loading experiment is shown in Figure 17, and the endpoint displacement increased with payload, as shown in Table 3. The prototype held 3.9 kg but not 4.0 kg, so the holding limit in this tested posture is bracketed between 3.9 and 4.0 kg. This posture-specific result is below the ideal 5.9 kg reference-posture prediction and is not a workspace-wide payload rating; the difference is consistent with the 24 V supply, current protection, manufacturing error, compliance, and the difference between nominal and available motor torque.

**Figure 18:**
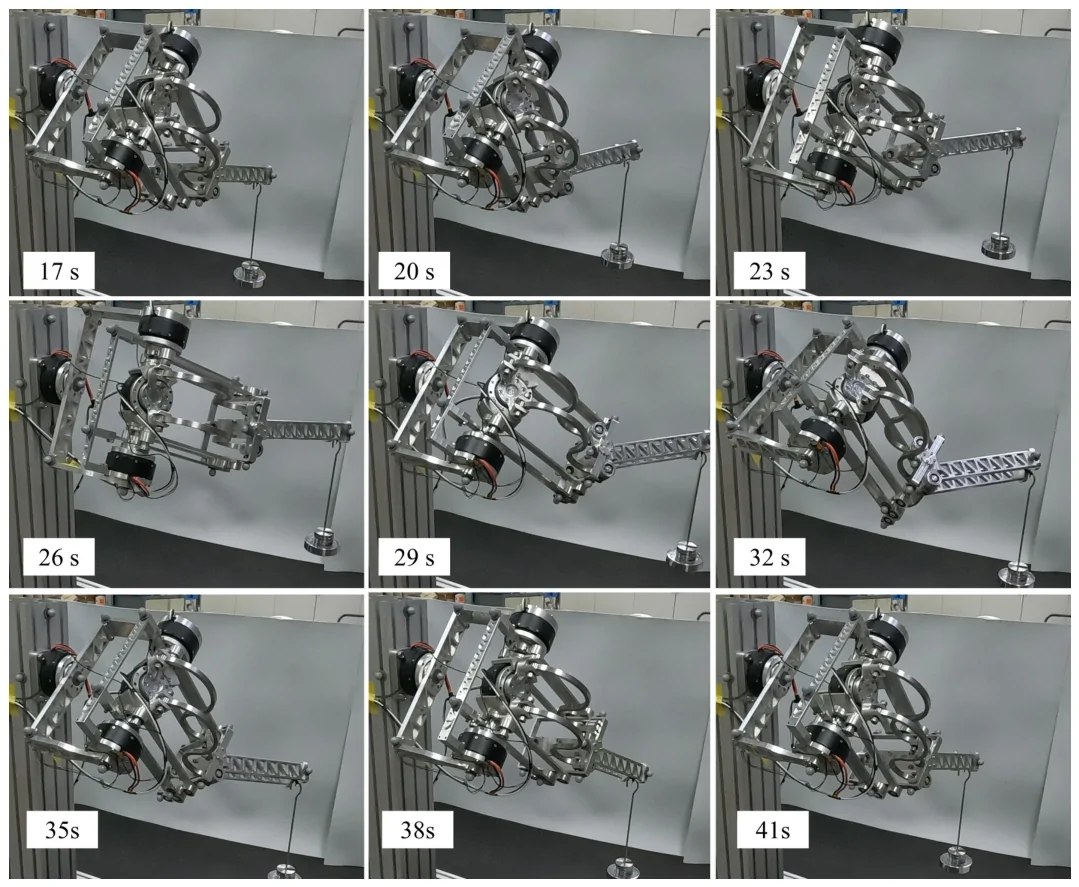
Prototype payload-tracking experiment with a 13 N load at the arm endpoint.

**Figure 19:**
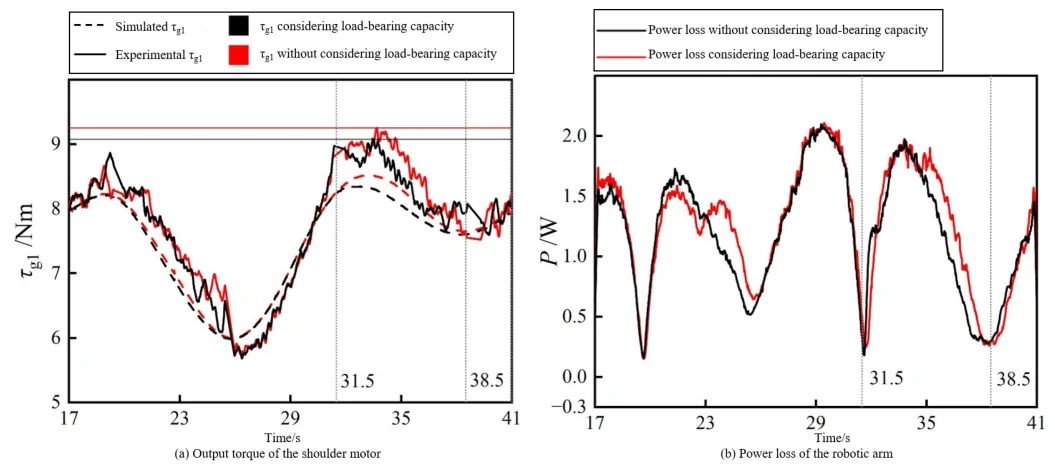
Prototype validation of the Case 1 trajectory. For the prototype Case 1 runs, the load-capacity-constrained trajectory shows a lower controller-recorded shoulder peak and a lower integrated/recorded positive mechanical energy value than the trajectory planned under geometric constraints only; these are descriptive comparisons for the tested condition.

Finally, the two trajectories from Simulation Case 1 were tested with a 13 N payload (approximately 1.33 kg), as shown in Figure 18. Case 1 was selected because it produced the largest simulated energy difference and included a segment in which the geometric-only trajectory exceeded the conservative planning torque limit; it was therefore the most discriminating of the two simulated cases. The 13 N load is well below the 3.9–4.0 kg posture-specific holding-limit bracket and was retained to provide a direct simulation-to-prototype comparison under the conservative 8.375 Nm planning limit. Each trajectory was executed three times, but the retained summary record does not contain the run-by-run values needed for defensible dispersion statistics. The joint tracking errors were mostly within ±0.1°, indicating that the joint controller followed the commands accurately. The shoulder motor produced the maximum torque. The reported shoulder peak was obtained from a 20-point neighborhood average around the return-torque peak. Figure 19 shows that this controller-recorded value was lower by approximately 1.83% in the tested runs. The recorded total-energy value decreased from 30.378 J to 28.85 J (5.03%). These values should not be interpreted as statistically resolved improvements because run-level data and measurement uncertainty are unavailable. Additional prototype datasets over multiple payloads, paths, speeds, and workspace regions were not available for this revision; the experiment is therefore a proof-of-principle validation, not a general performance envelope.

These prototype results verify that the planned posture trajectory can be executed under the tested condition. Their limited scope and the discrepancy from the ideal simulation are discussed in Section 5.

## 5. Discussion

The simulation and prototype results show that the arm angle is not only a geometric redundancy parameter but also a useful mechanical planning variable. By projecting both joint constraints and load-capacity constraints into the same one-dimensional domain, the planner can choose postures that are simultaneously feasible and mechanically favorable. This is particularly suitable for centrally driven humanoid arms because the load distribution among proximal actuators changes strongly with posture. Unlike manipulability optimization, which maximizes a local kinematic or force-transmission index, the proposed criterion directly tests whether each actuator can support a specified payload. Compared with predictive or general trajectory optimization, the present method is less general but reduces the posture search to a scalar feasible interval. The two approaches are complementary: manipulability, collision, or predictive dynamic costs could be optimized inside the geometric–mechanical feasible set developed here.

The results also reveal a substantial difference between simulation and prototype behavior. In Simulation Case 1, the energy decreased by 7.65 J, from 30.60 J to 22.95 J (25.0%), whereas the prototype saving was 1.528 J, from 30.378 J to 28.85 J (5.03%). Thus, the relative reduction differed by 19.97 percentage points. The Adams comparison represents an ideal rigid-body mechanism and mainly captures posture-dependent gravitational and payload work. The prototype additionally contains reducer friction, linkage and bearing losses, gearbox backlash, motor-driver and copper losses, current protection, structural deflection, and measurement noise. A substantial part of these losses is approximately common to both planned trajectories and therefore remains after posture optimization, diluting the percentage saving. Compliance and clearance also cause the executed endpoint and load moment arms to differ from the modeled values. The available experiment measured the combined actuator response and did not independently instrument each loss mechanism, so assigning a defensible percentage to each source is not possible from the present data. The quantitative decomposition above is consequently limited to the total ideal-versus-prototype difference; component-level loss identification is a subject for future experiments.

The prototype validation used one deliberately demanding trajectory and one payload level. The second simulated path shows that the method can produce a feasible solution along a different, larger-workspace path, although its energy reduction is smaller (4.49%) because the path contains less variation in load distribution. For other payloads, the qualitative behavior follows directly from the feasible-set definition: a lower required load enlarges $\Phi_{M}$ and makes the result approach geometric-only planning, whereas a higher load shrinks or can disconnect the feasible set. Near the payload boundary, modeling errors and structural deflection become more influential, so a safety margin or online load estimate is required. More prototype trials across payloads, speeds, and workspace regions are necessary before generalizing the measured reduction.

The present method is intended for static or slow payload manipulation, where gravity and external load dominate the joint torques. Rapid motion introduces inertial and Coriolis torques that can invalidate a static feasibility prediction. The formulation also assumes a known load magnitude and direction; payload uncertainty can shift the mechanical feasible boundary and increase planning error. In addition, collision detection, obstacle avoidance, force control, grasping, and hand dexterity are not addressed, and the current experiment concerns a four-degree-of-freedom shoulder–elbow positioning chain. Extension to arms with more degrees of freedom is possible by retaining the load-capacity inequality, but more than one redundancy parameter would produce a multidimensional feasible set and require a different search strategy.

Future work will identify reducer-friction and full dynamic parameters, introduce uncertainty margins and online payload estimation, and validate multiple trajectories and payload levels at different speeds. Hardware improvements will target link stiffness, machining accuracy, assembly clearance, and direct joint-torque or endpoint-force sensing. At the planning level, the load-capacity set will be combined with self-collision and obstacle constraints, and extended to multidimensional redundancy, predictive dynamic planning, and coordinated wrist/hand manipulation. A data-driven approximation may also reduce the computational cost of the dynamic model in real-time applications.

## 6. Conclusions

This paper proposed a load-capacity-constrained arm-angle planning method for a centrally driven humanoid robotic arm. Joint limits, singularity avoidance, and static payload capability are mapped into a common one-dimensional arm-angle domain, and a smooth trajectory is planned within their intersection. In the heavy-load simulation, the method kept joint torque below the conservative planning torque limit, reduced maximum output power by 13.3%, and reduced integrated positive mechanical energy from 30.60 J to 22.95 J (25.0%). In the tested large-workspace simulation, maximum torque decreased from 3.725 Nm to 3.456 Nm (7.22%) and integrated positive mechanical energy decreased from 119.88 J to 114.50 J (4.49%). In the single 13 N prototype condition, the controller-recorded shoulder peak was approximately 1.83% lower and the recorded energy value decreased from 30.378 J to 28.85 J (5.03%) while the target endpoint path was retained. These prototype values are descriptive because run-level statistics and acquisition metadata are unavailable. The results support the planning principle for the tested conditions but do not establish general torque or energy savings.

The conclusions are limited by the static rigid-body model, the use of a known downward payload, and prototype validation with one trajectory and one payload level. Reducer friction, compliance, backlash, fast-motion dynamics, environmental collisions, force control, and hand manipulation are not included. Future research should identify friction and dynamic parameters, improve structural stiffness and calibration, test multiple loads and workspace regions, incorporate uncertainty and online load estimation, add collision/obstacle and force-sensing constraints, and extend the formulation to manipulators with multidimensional redundancy.

## Figures and Tables

**Table 1:** Main physical parameters of the humanoid robotic arm.

Parameter	Value	Parameter	Value
Shoulder offset $L_{0}$	170 mm	Shoulder joint range	360°
Upper-arm length $L_{1}$	220 mm	Upper-arm joint range	210°
Forearm length $L_{2}$	293 mm	Elbow joint range	90°
Arm mass	6.1 kg	Forearm joint range	120°

**Table 2:** Mass units used in the static load-capacity model.

Unit	Mass (kg)	Unit	Mass (kg)
Unit 1	1.2057	Unit 3-3	1.1160
Unit 2	1.1451	Unit 4-0	0.7042
Unit 3-0	0.1806	Unit 4-1	0.2828
Unit 3-1	0.1513	Unit 4-2	0.2828
Unit 3-2	0.1513	Unit 4-3	0.1090

**Table 3:** Endpoint displacement under increasing payload in the static loading test. The first column is the load header, and the second row is labeled Displacement (mm).

**Load (kg)**	0	3.0	3.5	3.7	3.8	3.9	4.0
**Displacement (mm)**	5.76	27.35	31.40	33.16	33.92	34.35	–

## Data Availability

The data supporting the findings of this study are available from the corresponding author upon reasonable request.

## Abbreviations

The following abbreviations are used in this manuscript:

DOF	Degree of freedom
GC	Global configuration
IK	Inverse kinematics
MDH	Modified Denavit–Hartenberg
PD	Proportional–derivative
